# Characterization of the complete chloroplast genome of black soybeans, *Glycine max* (L.) Merr. (legume)

**DOI:** 10.1080/23802359.2019.1673227

**Published:** 2019-10-01

**Authors:** Liang Jin, Zhiping Wan, Dan Wei, Dawei Yin

**Affiliations:** a The Key Laboratory of Soil Environment and Plant Nutrition of Heilongjiang Province, Soil Fertilizer and Environment Resources Institute, Heilongjiang Academy of Agriculture and Science, Harbin, Heilongjiang, China;; b Nanchang Hongdu Hospital of Traditional Chinese Medicine, Nanchang, Jiangxi, China;; c Plant Nutrition and Resources Institute, Beijing Academy of Agriculture and Forestry Sciences, Beijing, China;; d College of Agricultural Science and Technology, Heilongjiang Bayij Agricultural University, Daqing, Heilongjiang, China

**Keywords:** Black soybeans, *Glycine max* (L.) Merr., Leguminosae, chloroplast genome, genetic evolution relationship

## Abstract

l.Black Soybeans (*Glycine max* (L.) Merr.) is a rare legume native for agricultural production by clearing toxins from the body in Chinese medicine. In this study, the chloroplast genome of *G. max* (L.) Merr. obtained is 151,212 bp in length as the circular. The overall GC contents of the cp genome are 35.3%. The phylogenetic maximum-likelihood tree was constructed on 18 species the family Leguminosae cp genomes. The result showed that *G. max* (L.) Merr. is closest related to Glycine soja in genetic evolution relationship. This complete cp genome of Black Soybeans will be important for agriculture in Northeast China

Black Soybeans, *Glycine max* (L.) Merr. have been widely consumed as food and as material for Oriental medicine for hundreds of years in Asia (Kim et al. 2008). Black soybean is merely a black variety of the soybean (Buzzell et al. 1987) and is a rare legume native to China and has been used in Chinese medicine to clear toxins from the body. It is considered a source of balance, which has therapeutic effects include of replenishes blood levels, nourishing yin, expels wind, strengthens weakened, and normalizes the activity of the channels of stomach and spleen (Li et al. 2008). To study the family Legume species genetic evolution and medicinal value, we choose to study the chloroplast (cp) genome of Black Soybeans (*G. max* (L.) Merr.) as a representative. Here, the complete cp genome of *G. max* (L.) Merr. was obtained and researched, which will be very important for sustainable agricultural production and medicinal value in northeastern China and further.

The specimen sample of *G. max* (L.) Merr. was collected from test field of Heilongjiang Academy of Agriculture and Science in Harbin, Heilongjiang, China (126.62E; 45.69 N). The total cp DNA of *G. max* (L.) Merr. was extracted using the optimized CTAB method and stored in Soil Fertilizer and Environment Resources Institute, Heilongjiang Academy of Agriculture and Science, The Key Laboratory of Soil Environment and Plant Nutrition of Heilongjiang Province (No. SFERI-HAAS-01). The total genomic DNA was used for the shotgun library construction and sequenced using the Illumina HiSeq 4000 Sequencing Platform System (Illumina Co., San Diego, CA). Quality control was performed to remove low-quality reads and adapters using the FastQC (Andrews 2015). The cp genome was assembled using the Plann (Huang and Cronk 2015) and annotated using the MitoZ (Meng et al. 2019). The tRNA genes were further identified using Blast and tRNAscan-SE v2.0 (Lowe and Chan 2016). The physical map of the cp genome was generated using OrganellarGenomeDRAW v1.3.1 (Greiner et al. 2019).

The complete cp genome of *G. max* (L.) Merr. is 151,212 bp in length and has the circular molecular structure, containing a large single-copy region (LSC) of 83,096 bp, a small single-copy region (SSC) of 17,978 bp, and a pair of inverted repeat regions (IRs) of 25,069 bp each. The overall nucleotide composition is: 32.4% of A, 32.3% of T, 17.4% of C, and 17.9% of G, with the total GC content of 35.3%. The cp genome of *G. max* (L.) Merr. comprised 128 genes, including 37 protein-coding genes (PCGs), 37 transfer RNA (tRNA) genes, and 8 ribosomal RNA (rRNA) genes. In each one of the IR regions, 18 genes were found duplicated, which includes 7 PCG species (*rpl2*, *rpl23*, *ycf2*, *ndhB*, *rps7*, *rps12*, and *ycf1*), 7 tRNA species (*trnI-CAU*, *trnL-CAA*, *trnV-GAC*, *trnI-GAU*, *trnA-UGC*, *trnR-ACG*, and *trnN-GUU*), and 4 rRNA species (*rrn16*, *rrn23*, *rrn4.5*, and *rrn5*). The accurate annotated cp genome has been submitted to NCBI GenBank and the accession number is MK9478681.

The neighbour-joining (NJ) analysis was applied to the 18 species cp genomes based on amino acid sequences of 25 PCGs. The phylogenetic tree was reconstructed using NJ methods and performed using the RaxML v8 (Stamatakis 2014), which the bootstrap value was calculated using 5000 replicates to assess node support and all the nodes were inferred with strong support by the NJ methods. The genetic evolutionary tree was constructed using the MEGA X (Kumar et al. 2018) and edited using the iTOL v4 (Ivica and Peer 2019). The phylogenetic tree of these cp genomes showed that *G. max* (L.) Merr. cp genome is evolutionarily closest to that of *Glycine soja* (NC_022868.1) (Figure 1). The result presented here will contribute to the further biological study into the family Legume plants and is also important for sustainable agricultural production and medicinal value in northeast China and further.

**Figure 1.**
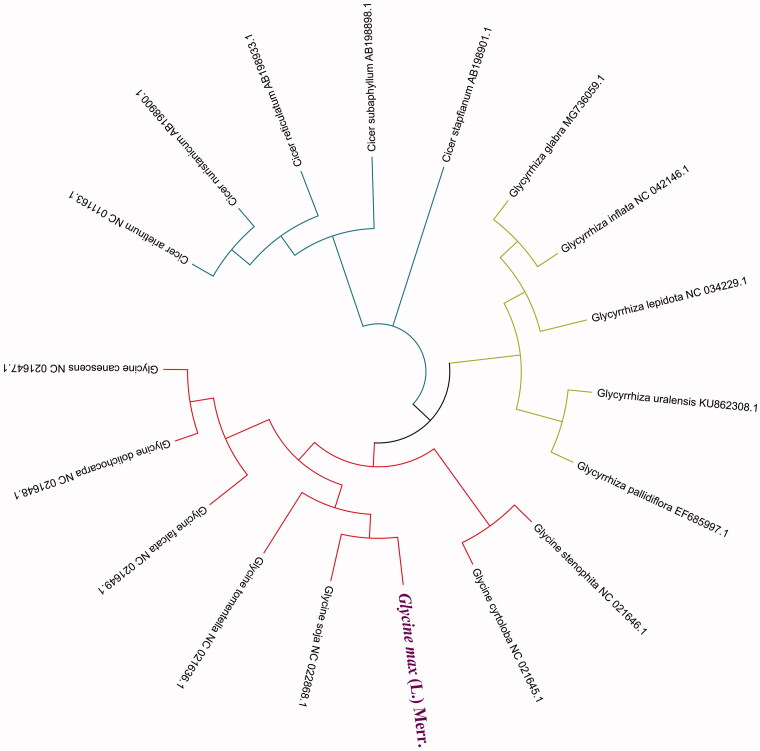
Neighbour-joining method of 18 species the family Leguminosae chloroplast genomes based on amino acid sequences of 25 PCGs. Numbers above each node represent bootstrap values from 5000 replicates.
